# Preparing Laboratories for Interconnected Health Care

**DOI:** 10.3390/diagnostics11081487

**Published:** 2021-08-17

**Authors:** Andreas Bietenbeck, Thomas Streichert

**Affiliations:** 1 Institut für Laboratoriumsmedizin, Medizinische Mikrobiologie und Technische Hygiene, München Klinik, 80804 Munich, Germany; 2 Institut für Klinische Chemie, Universität zu Köln, Medizinische Fakultät und Uniklinik Köln, 50937 Cologne, Germany; thomas.streichert@uk-koeln.de

**Keywords:** interconnected healthcare, LOINC, SNOMED-CT, UCUM, NPU

## Abstract

In an increasingly interconnected health care system, laboratory medicine can facilitate diagnosis and treatment of patients effectively. This article describes necessary changes and points to potential challenges on a technical, content, and organizational level. As a technical precondition, electronic laboratory reports have to become machine-readable and interpretable. Terminologies such as Logical Observation Identifiers Names and Codes (LOINC), Nomenclature for Properties and Units (NPU), Unified Code for Units of Measure (UCUM), and SNOMED-CT can lead to the necessary semantic interoperability. Even if only single “atomized” results of the whole report are extracted, the necessary information for correct interpretation must be available. Therefore, interpretive comments, e.g., concerns about an increased measurement uncertainty must be electronically attached to every affected measurement result. Standardization of laboratory analyses with traceable standards and reference materials will enable knowledge transfer and safe interpretation of laboratory analyses from multiple laboratories. In an interconnected health care system, laboratories should strive to transform themselves into a data hub that not only receives samples but also extensive information about the patient. On that basis, they can return measurement results enriched with high-quality interpretive comments tailored to the individual patient and unlock the full potential of laboratory medicine.

## 1. Introduction

Laboratory medicine has a long history of using information technology to optimize processes in the laboratory. Now information and telecommunication technologies promise to transform the whole of medicine. In an interconnected health care system, a team of specialists replaces the single physician who is solely responsible for a patient. In fragmented clinical pathways, telecommunication links specialized entities. Here, laboratory measurements of a patient are no longer conducted in a single laboratory. Even today, rare analyses are usually conducted in specialized laboratories. Point-of-care tests, including continuous sensors, perform additional measurements. Using electronic patient records or other IT infrastructure, laboratory results will be exchanged and aggregated. Transmitted electronically, they are always available at the point of treatment. Various medical specialists are no longer the only recipients of the laboratory report. Other entities, such as machine learning algorithms, receive laboratory reports electronically to predict diseases or disease progression [1]. Other programs will evaluate previous results to inform on retest intervals and to prevent unnecessary testing [2].

Because laboratory reports will be readily available, they will be used in other contexts besides immediate patient treatment. For hospital management and resource allocation, laboratory results already enter dedicated scores [3]. Laboratory data is also analysed in large health care research projects [4]. The COVID pandemic provides examples where test results are aggregated to influence public health decisions. Patients see themselves in a more active role and request their laboratory reports to manage their lifestyle and health.

This work examines how laboratories and laboratory medicine can prepare for these changes on a technical, content, and organizational level.

## 2. Materials and Methods

A comprehensive literature review was conducted.

## 3. Results

### 3.1. International Terminologies Allow Machine-Readable, Unambiguous Designation of Laboratory Analyses

Technically, laboratory reports must be transferable, machine-readable, and well-structured for a safe and efficient exchange. Established terminologies for different parts of the laboratory report enable semantic interoperability and allow correct interpretation from all stakeholders in a connected healthcare process.

Even in a single country, laboratory analyses usually do not have the same name. To give an illustrative example, Vitamin D is named “Calcidiol”, “Calciol”, “25-OH-Vitamin D”, “Vitamin D3”, “25-Hydroxycholecalciferol” or “Cholecaliferol” and is typically reported in at least two different units, “ug/L” and “mmol/L”. Human recipients of laboratory reports may therefore struggle to recognize related measurements from different labs. For software interpreting laboratory measurements, unambiguous identifiers are an indispensable prerequisite. Internationally, two terminologies, by name the Logical Observation Identifiers Names and Codes (LOINC) and the Nomenclature for Properties and Units (NPU), are in use. Briefly, LOINC assigns a unique identifier to a fixed combination of six parts: Component (the measured analyte), Property (measured quantity of the analyte, e.g., mass), Time Aspect (moment in time or a time interval, e.g., 24-h urine), System (sample material, e.g., serum), Scale (level of measurement e.g., quantitative), and Method (often optional). NPU is predominantly used in northern Europe and uses only three axes: System (sample material), Component (measured analyte), and Kind-of-Property (quantity that is measured, e.g., amount-of-substance concentration). NPU is heavily influenced by metrological concepts and provides unique identifiers for laboratory analyses. LOINC provides more but not all information (e.g., different methods) that might influence the comparability of laboratory results [5].

Besides the laboratory analysis, the unit of the result has to be machine-readable for interconnection and exchange of laboratory results. The Unified Code for Units of Measure (UCUM) system provides an easy syntax to express all common units. It is not necessary to prefer International System of Units (SI) units to traditional units because with UCUM software should be able to convert both according to the recipient’s needs.

Non-numeric results, such as a bacterium identified in blood culture, require other terminologies such as SNOMED-CT. For reports containing genetic information, the variants are often encoded using the Online Mendelian Inheritance in Man catalogue (OMIM) [https://www.omim.org, accessed 16 August 2021]. Interpretive comments should be encoded again using standardized terminology as much as possible. SNOMED CT and the international classification of diseases (e.g., ICD-10/11) [https://www.who.int/standards/classifications/classification-of-diseases, accessed 16 August 2021] offer many appropriate terms. However, electronic data standards, such as Fast Healthcare Interoperability Resources (FHIR,) offer well-standardized resources for the plain laboratory report but are less well developed for interpretative comments.

Despite considerable efforts for standardization and harmonization, the measuring device influences some results considerably. Device-specific target values exist in many external quality assessment (EQA) schemes. In internal quality control materials, assayed targets account for the device. Knowledge of the measuring device is therefore often required for correct interpretation of some analyses (e.g., tumor markers). Therefore, it is important to encode the device unambiguously and consistently in electronic laboratory reports. The Global Unique Device Identification Database (GUDID) by the U.S. Food and Drug Administration contains unique keys identifying medical devices, including measuring devices. The European Union constructs a similar database (EUDAMED) for this purpose. Laboratories can use the enclosed keys and identifiers not only of their measuring devices but also of their test kits used to include this information in their electronic laboratory reports. This strategy offers a new quality of metrological transparency and helps to prevent the erroneous cumulation of incommensurable results.

Many laboratories organize point-of-care testing for their hospitals. Sufficient education of all POCT operators is mandatory. However, as these operators on the wards are no laboratory professionals, user errors might occur more often than in the core laboratory [6]. The type of operator layperson, medical professional, or laboratory professional should therefore be included in the electronic laboratory reports as well.

To increase awareness, some laboratories communicate special results only as part of their comments instead of in their standard result field. This approach hinders machine interpretation. Results should always be written in the appropriate field. Comparators (less than “<”, greater than “>”) can be used when the exact value cannot be determined e.g., at the limit of quantification. In addition, comments can express increased uncertainty.

### 3.2. Reports Must Be “Atomizable” into Single Measurements without Loss of Information

In many settings in an interconnected health care system, only single measurements and not the whole diagnostic report are processed. For example, a clinical decision support system warning of acute kidney injury may only use creatinine measurements and not the whole report. To allow such applications a report must be “atomizable” into single measurements without loss of information that is relevant for these measurements. Revisiting the creatinine example, any comment describing an increased measurement uncertainty must be electronically attached to the measurements. With this information, the decision support system can react appropriately.

Some data formats, such as FHIR, are modular and allow selecting single measurements. Other formats are more document-oriented. Because only whole laboratory report and not separate parts can be retrieved these formats may generating more overhead. Regardless of the format, all the required information for interpretation has to be stored. As a rule, the less the measurement is standardized the more information is required.

### 3.3. Interpretive Comments Are a Crucial Part of Electronic Laboratory Reports

Besides the measurement results, interpretative comments are a crucial part of electronic laboratory reports. They can be grouped into at least four categories. First, truly interpretive comments usually summarize many measurements and explain a likely condition of the patient or point directly to a diagnosis, sometimes combined with recommendations for action. These comments are the analogue of interpretations from other medical diagnostic disciplines. Second, warnings indicating that a particular measurement can only be conducted with higher uncertainty. Usually, preanalytical influences, interferences, or less than ideal sample conditions require this kind of comment. If possible, the higher uncertainty should be quantified. These comments are of vital importance for correct interpretation by humans or computerized systems alike. In extreme cases, a high uncertainty might invalidate the whole measurement. Unfortunately, to the best of our knowledge, no electronic standard has been developed to store and transmit this information. Third, administrative comments communicate organizational directions and notes to the recipient of the laboratory report. Related comments concern information on test appropriateness [2]. These comments are of little importance in an interconnected health care system because most of the recipients of the laboratory report are no longer responsible for organizational issues and test ordering. Laboratories should evaluate other forms of communication for these messages. Fourth, flags based on e.g., reference limits, clinical decision limits, or therapeutical limits are added to the report, sometimes to point out extreme values. These flaggings are not standardized and show a high variation between laboratories, starting from the character(s) applied for flagging and ending with strategies such as multilevel flagging [7]. Since these flags are widely used in laboratory reports, there is a strong need for standardization.

The demand for high-quality interpretive comments is likely to rise. Nowadays, a typical laboratory offers hundreds of different tests and as the spectrum of analytical procedures and tests still increases, expert interpretation of laboratory data is more likely to become a vital part of the report. Since up to 80% of clinical decisions are affected by in vitro diagnostics [8], these expert interpretations can improve patient safety, timely diagnosis, and treatments [9].

### 3.4. Interconnectivity Requires Traceable Measurements

If laboratory results are exchanged in interconnected systems and ultimately transferred to the electronic patient archive, several different laboratories will conduct measurements of the same analyte, e.g., TSH in the hospital laboratory and follow-up in general practitioner/ambulant laboratories. To interpret these measurements from diverse sources correctly, all measurements should be traceable to metrological standards. In addition, metrologically traceable measurements facilitate a safer and more widespread application of scores and electronic assistance tools such as clinical decision support systems. When employing these instruments in a new setting, they still have to be tested and extensively recalibrated to account for local characteristics, among them analytical differences of laboratory measurements [10,11,12,13]. This approach hinders progress in medicine and will be unfeasible if electronic tools become more commonplace.

ISO 15189 already asks laboratories to estimate measurement uncertainty for their analysis. Theoretically, measurement uncertainty can help interpretation by humans and computerized systems. The Guide to the Expression of Uncertainty in Measurement (GUM) [14] offers some well-tested principles. However, the debate on how to estimate measurement uncertainty is still ongoing in clinical chemistry [14,15,16]. Every consensus should differentiate between traceable measurands controlled with commutable quality control sample and analyses, that are less well standardized and where stability is controlled with quality control samples with assayed targets or consensus means. As an example, glucose measurements are well standardized. Here, measurement uncertainty can help to decide whether the total testing process of different laboratories leads to comparable performance. On the other hand, for many tumour markers, no agreed-upon traceable standard exists and measurements differ substantially depending on the manufacturer of the measurement device. In these cases, the measurement uncertainty can only be expressed with regard to the measurement of the specific manufacturer. The measurand should be named accordingly (e.g., CA 19-9 (manufacturer X)), and the unique device identifier, as well as the instrument type, should be included).

Of course, IVD manufacturers and laboratories are far from a full standardization or even harmonization. In this situation, the information about bias and imprecision is crucial. The use of method-specific reference intervals (RIs) or, for research, normalization of test results [17,18], are interesting and could help to facilitate the comparison of measured values from different sources. The weakness of the reference interval-based approach is caused by a lack of standardized reference interval estimation procedures. Some laboratories estimate reference intervals from routine laboratory results (indirect reference interval estimation), few laboratories carry out direct studies to estimate RIs and most of the laboratories employ reference intervals derived from scientific literature or the IVD manufacturer. Reference intervals include the biological variation and the variability of the underlying measurement procedures and are therefore unsuitable for standardization [19]. Nevertheless, normalization strategies such as percentile transformation [20] offer interesting methods to compare results from different laboratories.

### 3.5. Opportunities Inside the Laboratory

At present only a few applications exist in laboratories that use data mining strategies or AI for diagnostics. Commercially available products focus on picture recognition techniques e.g., in microscopic processes for cell classification [CellaVison–DiffMaster Octavia Haematology Analyzer, https://www.accessdata.fda.gov/cdrh_docs/pdf/K003301.pdf, accessed 16 August 2021]. The lack of AI or deep learning algorithms in medicine is amongst other reasons caused by non-categorized, non-standardized, and incomplete data. In addition, clinical data is only available for few researchers, typically in scientific surroundings such as university hospitals. If laboratories meet the aforementioned preconditions, the prerequisites are in place for exchanging, combining, and analysing standardized and complete data in machine-readable formats. This could enable and accelerate AI developments and offers, besides scientific progress, the option for clinical improvements.

### 3.6. Laboratories Need to Engage All Stakeholders

This work has argued that in an interconnected age of medicine, laboratory reports must contain all relevant information. Correct interpretation must be possible without additional information, e.g., contacting the laboratory personally. Because of complex and sometimes fragmented clinical pathways and greater specialization, the danger of increasing the distance between laboratory and treating physicians must be addressed on an organizational level. Laboratories need to maintain a close relationship with the treating physician to remain part of the health care team and not to become an anonymous entity. Only laboratory specialists engaged in individual patient care can convince clinicians and reach an appropriate use of laboratory diagnostics [21].

To meet the needs of laboratory medicine in interconnected medical systems, External Quality Assessment should increasingly focus on international standardization or harmonization. If a certified reference material cannot be used, target values should be assigned with a reference measurement procedure. Sample material should always be commutable and resemble the behaviour of real patient samples [22]. Internal quality control strategies should take the traceability of calibrators and control materials into account [23].

For many laboratory tests, a meaningful interpretation depends on additional extra-analytical information. In therapeutic drug monitoring, target concentrations for many immunosuppressants vary by the transplanted organ [24]. When integrating genomics, proteomics and other high-dimensional data with clinical information prognostic models are usually improved [25]. In interconnected health care settings, laboratories should strive to receive not only samples and measurement requests but also additional clinical information to enhance the value of the results. To maintain high efficiency, this information should be transmitted electronically.

Similarly, high-quality interpretive comments often require the integration of laboratory results with other clinical data. When laboratories form only an isolated node in larger patient pathways, they risk missing this crucial information. However, easy electronic information exchange in an interconnected age of medicine should enable laboratories to receive the necessary clinical data. In these networks, the role of laboratories expands from data provider to data hub. Laboratories need to maintain and develop the necessary expertise further for providing high-quality interpretative comments. To ensure high efficiency, they also need to improve IT skills to extract and process the relevant clinical data and to attach interpretive comments electronically.

**Figure 1:**
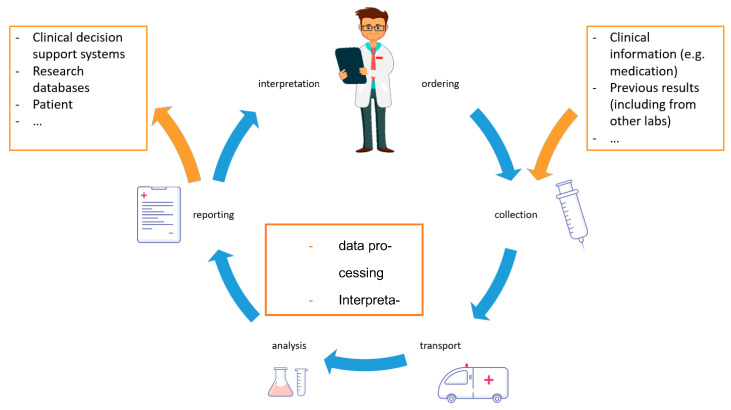
The classical brain-to-brain loop [27] describing the total testing process from the selection of the test to the return of the final result is expanded in interconnected health care systems. The laboratory electronically receives additional information besides the test order. On this basis, the laboratory can generate high-quality interpretations and comments of the results. The recipient of the report is no longer the treating physician but also electronic applications and patients themselves.

The traditional brain-to-brain loop [26] with the physician ordering a test and receiving the result has to be extended (Figure 1). In interconnected health care systems, the number of recipients of laboratory reports multiplies and the required information accompanying a sample and measurement request increases as well. At the interfaces, the role of information and telecommunication technologies is likely to increase. Other high-dimensional analyses require profound software and data analytic competencies. Laboratories need to further develop the relevant skills to realize the full potential of their analyses for patient treatment.

## 4. Discussion

In interconnected medicine, laboratory reports are exchanged in complex patient pathways. Various specialists and computer algorithms interpret the analytical results for diagnosis and patient care. Some of the described changes are hypothetical but many beginnings of interconnected health care systems in medicine are already visible. This work describes how individual laboratories, as well as the whole of laboratory medicine, have to adjust to these transformations to improve patient care.

The described changes require considerable efforts by the laboratory. Based on high-quality (and ultimately standardized) laboratory test procedures and the profound knowledge of laboratory medicine, the sending of electronic laboratory reports containing all the required information if necessary, combined with an interpretation, is becoming a vital part of patient care. The establishment and maintenance of electronic laboratory reports with semantically and syntactically interoperable standards can be challenging. Traceable measurements and tighter analytical performance specifications certainly will be more expensive. These costs have to be borne by all parties benefitting from these improvements.

This work has argued that laboratory reports have to be annotated to allow correct interpretation. The recent advances in machine learning seem to suggest that these algorithms can handle even less well-structured laboratory reports without further annotations. However, while the determination of the exact sample size is still open to research, it seems certain that less structure will require more samples to reach the same performance [28]. Structured data will also help to generate information that is more trustable [29].

Last but not least, a central stakeholder is the patient: if laboratories fulfil the above-mentioned requirements, it is also possible to include the patient in the communication. This could start with adding information to the order by the patient, such as dietary or lifestyle habits and could lead to an electronic report which could be sent to the patient, e.g., via smartphone apps [30,31]. Especially for diseases with a close relationship between laboratory test results and the adjustment of therapy, e.g., diabetes, a number of possible applications exist that could complement diabetes care [32].

As described here, laboratories can transform into a hub in an interconnected health care system that receives samples, measurement requests, and additional information and generates measurement results and interpretations meaningful for a wide audience. The required IT skills at the interfaces and for automated comment generation are non-trivial. It can be argued that these skills are outside of the core competencies of a laboratory. Indeed, further specialization could reduce medical laboratories to mere high throughput measuring centres, but if laboratories take on the task they can even improve their central role in diagnostics for the benefit of patients.

## Data Availability

Not applicable.
